# Methylation Analysis of *P16, RASSF1A, RPRM,* and *RUNX3* in Circulating Cell-Free DNA for Detection of Gastric Cancer: A Validation Study

**Published:** 2020

**Authors:** Kioomars Saliminejad, Shahrzad Soleymani Fard, Hamid Reza Khorram Khorshid, Marjan Yaghmaie, Habibollah Mahmoodzadeh, Seyed Asadollah Mousavi, Seyed Hamidollah Ghaffari

**Affiliations:** 1. Hematology, Oncology and Stem Cell Transplantation Research Center, Shariati Hospital, Tehran University of Medical Sciences, Tehran, Iran; 2. Reproductive Biotechnology Research Center, Avicenna Research Institute, (ACECR), Tehran, Iran; 3. Genetics Research Center, University of Social Welfare and Rehabilitation Sciences, Tehran, Iran; 4. Department of Surgery, Cancer Institute, Imam Khomeini Hospital, Tehran, University of Medical Sciences, Tehran, Iran

**Keywords:** Biomarkers, Cell-free DNA, Gastric cancer, DNA methylation

## Abstract

**Background:**

Most of Gastric Cancer (GC) patients are diagnosed at an advanced stage with poor prognosis. Hypermethylations of several tumor suppressor genes in cell-free DNA of GC patients have been previously reported. In this study, an attempt was made to investigate the methylation status of *P16, RASSF1A, RPRM,* and *RUNX3* and their potentials for early diagnosis of GC.

**Methods:**

Methylation status of the four tumor suppressor genes in 96 plasma samples from histopathologically confirmed gastric adenocarcinoma patients (Stage I–IV) and 88 healthy controls was determined using methylation-specific PCR method. Receiver operating characteristic curve analysis was performed and Area Under the Curve (AUC) was calculated. Two tailed p<0.05 were considered statistically significant.

**Results:**

Methylated *P16, RASSF1A, RPRM*, and *RUNX3* were significantly higher in the GC patients (41.7, 33.3, 66.7, and 58.3%) compared to the controls (15.9, 0.0, 6.8, and 4.5%), respectively (p<0.001). Stratification of patients showed that *RPRM* (AUC: 0.70, Sensitivity: 0.47, Specificity: 0.93, and p<0.001) and *RUNX3* (AUC: 0.77, Sensitivity: 0.59, Specificity: 0.95, and p<0.001) had the highest performances in detection of early-stage (I+II) GC. The combined methylation of *RPRM* and *RUNX3* in detection of early-stage GC had a higher AUC of 0.88 (SE=0.042; 95% CI:0.793–0.957; p<0.001), higher sensitivity of 0.82 and reduced specificity of 0.89.

**Conclusion:**

Methylation analysis of *RPRM* and *RUNX3* in circulating cell free-DNA of plasma could be suggested as a potential biomarker for detection of GC in early-stages.

## Introduction

Gastric Cancer (GC) is the third leading cause of cancer-related mortality globally 1. Diagnosis of early-stage GC is still difficult, and in fact, most of them are diagnosed at an advanced stage with a poor prognosis 2. Currently, invasive endoscopy followed by pathological diagnosis is the gold standard for GC diagnosis 3. Single-lesion tumor-biopsy could not reflect the tumor heterogeneity, which could result in the treatment failure and drug resistance 4. In addition, the low sensitivity and specificity of available blood biomarkers are not satisfactory for early diagnosis of GC 2.

Epigenetic changes, including aberrant DNA methylation, are common in all types of cancers including gastrointestinal, and contribute to both initiation of cancer and progression 5. Deregulation of epigenetic modifications may actually even precede classical genetic changes in various oncogenes and tumor suppressor genes 6. Aberrant DNA methylation is not just a feature of advanced-stage, but also an early and driver event in GC 7, and could be non-invasively identified in cell-free DNA (cfDNA) of cancer patients 5.

Aberrant methylation of several tumor suppressor genes including *RUNX3*, *P16*, *RASSF1A*, *ZIC1*, *RPRM*, *CDH1*, and *SOX17* as potential biomarkers for early detection of GC has been identified 8. However, to become a clinically approved test, a potential biomarker should be confirmed and validated in inter- and intra-laboratory studies using hundreds of specimens 9. The first step in finding a biomarker usually begins with studies of tumor tissues and non-tumor tissues 10. Previous studies showed that the methylation of *P16* 11,12, *RASSF1A* 13, *RPRM* 14, and *RUNX3* 11 was significantly higher in primary GC tissues compared to the corresponding normal gastric tissues.

*P16,* a cell cycle regulator, controls the G1 phase of cell cycle to S phase, and inhibits CDK4 and CDK6 15. *RASSF1A*, a putative tumor suppressor gene, plays an important role in regulation of cell cycle, apoptosis, and microtubule stability through the regulation of Ras signaling 16. *RPRM* in response to p53 expression arrests cell cycle at G2/M, and its expression is inversely associated with the cell proliferation and growth in GC 17. *RUNX3* is a tumor suppressor gene considered as a downstream effector of the TGF-β signaling pathway 18.

Previous studies using the serum or plasma samples have shown that methylation of the *RASSF1A* 19, *RUNX3* 20, *RPRM* 21,22, and *P16* 23, as potential diagnostic biomarkers, could be suggested for early detection of GC*.* In this study, an attempt was made to investigate and validate the potential of these tumor suppressor genes as diagnostic biomarkers in the plasma of GC patients and normal controls.

## Materials and Methods

### Subjects

Altogether, 184 plasma samples from 88 normal healthy controls, and 96 histopathologically confirmed gastric adenocarcinoma patients with various Tumor-Node-Metastasis (TNM) stages (I–IV) were collected. The plasma samples were collected prior to surgery, chemotherapy, and/or radiotherapy. Participants were enrolled from the Department of Surgery, Cancer Institute, Tehran University of Medical Sciences, Tehran, Iran. The patients were followed-up until death or to the end of the study. The study was approved by the ethics committee of the Tehran University of Medical Sciences (Ethics code: IR.TUMS.VCR.REC.1395. 1078), and written informed consent was obtained from all patients.

### DNA extraction from plasma samples

From each participant, 5 *ml* of peripheral blood was collected in 200 *uL* of 0.5 *M* EDTA. To separate the plasma, the blood samples were immediately centrifuged at 3000×*g* for 10 *min* at 4*°C*. The plasma was collected and transferred to new tubes and stored at −80*°C*. Circulating cfDNAs was extracted from 2 *ml* of plasma samples by the QIAamp Circulating Nucleic Acid Kit (Qiagen, Germany) according to the manufacture’s protocol. The concentrations of cfDNAs were measured using a NanoDrop ND-1000 spectrophotometer (NanoDrop Technologies, Wilmington, DE, USA). For all samples, before proceeding to sodium bisulfite conversion step, the accuracy of cfDNAs extractions were assessed by amplifying *TBP* (TATA-binding protein) gene using the forward 5′-CACAGACTCTCACA ACTGCAC-3′ and reverse primer 5′-ACAATCCCAG AACTCTCCGTAG-3′. The 115 *bp* PCR products of the *TBP* housekeeping gene were amplified in all cf- DNAs extracted in the plasma of GC patients as well as control samples.

### Sodium bisulfite conversion

For each sample, 40 *uL* of cfDNA was modified with sodium bisulfite using the EpiTect Bisulfite Kit (Qiagen, Germany) according to the manufacture’s protocol of sodium bisulfite conversion of unmethylated cytosines in DNA from low-concentration solutions. Purified modified DNA was eluted in 30 *μL* of DDW and sorted at −80*°C* until use.

### Methylation specific PCR

**Figure 1.**
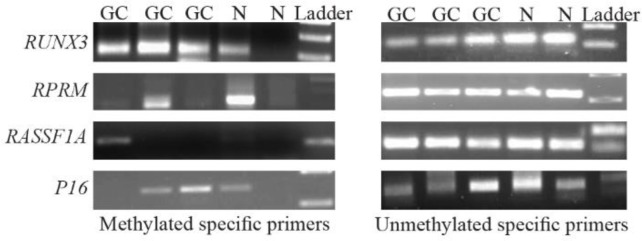
Agarose gel electrophoresis of methylation specific PCR products for *RUNX3*, *RPRM*, *RASSF1A*, and *P16* in the plasma samples of gastric cancer (GC) patients and the controls (N). Met: Methylated-specific primers; UnMet: Unmethylated-specific primers. *RUNX3* Met (115 *bp*); *RUNX3* Unmet (115 *bp*); *RPRM* Met (120 *bp*); *RPRM* Unmet (126 *bp*); *RASSF1A* Met (102 *bp*); *RASSF1A* Unmet (108 *bp*); *P16* Met (147 *bp*); *P16* Unmet (149 *bp*).

The methylation status of the *P16, RASSF1A, RPRM,* and *RUNX3* promoters in cfDNA samples was detected by conventional methylation specific PCR (MSP) by specific primer pairs for both the methylated and unmethylated status (Table 1). Each MSP reaction was performed in a total volume of 12 *uL*. Briefly, 2 *uL* of sodium bisulfite converted DNA was added into a reaction mixture containing 6 *uL* of 2x master mix (Ampliqon, Denmark), and 1 *uL* of the corresponding forward and reverse primers (10 *uM*); finally ddw was added to a final volume of 12 *uL*. Amplification conditions for both methylated and unmethylated reactions were as follows: an initial denaturation of 95*°C* for 5 *min*, followed by 45 cycles of 95*°C* for 35 *s*, annealing temperature of 57–67*°C* for 35 *s*, and extension of 72*°C* for 35 *s*, and finally an extension of 72*°C* for 10 *min*. The MSP products were electrophoresed on 2% aga-rose gels, stained with ethidium bromide, and visualized under UV light (Figure 1).

**Table 1.** Methylated and unmethylated specific primer pairs used in methylation specific PCR

**Gene**	**Forward primer 5′→3′**	**Reverse primer 5′→3′**	**Tm F/R**	**Product length (*bp*)**
***P16***				
Met	ATTAGAGGGTGGGGCGGATCGC	ACCCCGAACCGCGACCGTAA	67/67	147
Unmet	ATTAGAGGGTGGGGTGGATTGT	CAACCCCAAACCACAACCATAA	61/60	149
***RASSF1A***				
Met	GTTGGTATTCGTTGGGCGC	AACTACCGTATAAAATTACACGCG	59/58	102
Unmet	GGAGTTGGTATTTGTTGGGTGT	ACCAACTACCATATAAAATTACACACA	58/57	108
***RPRM***				
Met	TGCGAGTGAGCGTTTAGTTC	CTAATTACCTAAAACCGAATTCATCG	58/58	120
Unmet	AGTTTGTGAGTGAGTGTTTAGTTT	ATCTAATTACCTAAAACCAAATTCATCA	57/57	126
***RUNX3***				
Met	ATAATAGCGGTCGTTAGGGCGTCG	GCTTCTACTTTCCCGCTTCTCGCG	65/66	115
Unmet	ATAATAGTGGTTGTTAGGGTGTTG	ACTTCTACTTTCCCACTTCTCACA	57/59	115

The underlined nucleotides indicated the CpG sites.

### Statistical analysis

Statistical analysis of the data was performed using the SPSS version 16.0 (SPSS Inc, Chicago, IL). The methylation status and the other qualitative variables were expressed as frequencies and percentages. Continuous variables were compared by Student’s t test, while categorical data were checked by Chi-square or Fisher’s exact tests where appropriate. Receiver Operating Characteristic (ROC) curve and the Area Under the Curve (AUC) were used to assess the performance-of the biomarkers and the higher AUCs were considered as better diagnostic performance. A logistic regression model was performed to evaluate the diagnostic performance of the combination of the biomarkers. Survival rates were calculated with the Kaplan-Meier method and the statistical difference between survival curves was determined with log-rank test. Two tailed p-value <0.05 was considered statistically significant.

## Results

### Descriptive analysis of the subjects

The mean age of GC patients and healthy controls were 59.5±12.3 and 56.1±11.3, respectively. The female to male gender ratio in the control and GC groups were 26/62 and 34/62, respectively. No significant difference was found in the distribution of age (p=0.052) and gender (p=0.434) among the GC patients and the normal healthy controls. In the GC group, the ratio of males (n=62) to females (n=34) was nearly twice. Classification of the GC patients according to the TNM classification showed that 35.4% (34/96) and 64.6% (62/96) of the tumors were early (I+II) and advanced-stages (III+IV), respectively.

### Methylation rates and performances of the candidate genes

The *P16, RASSF1A, RPRM,* and *RUNX3* promoters were found to be methylated in 40 (41.7%), 32 (33.3%), 64 (66.7%), and 56 (58.3%) of the 96 GC samples, respectively. Alternatively, the *P16, RASSF1A, RPRM,* and *RUNX3* promoters were found to be methylated in 14 (15.9%), 0 (0.0%), 6 (6.8%) and 4 (4.5%) of the 88 control samples, respectively (Table 2). Unmethylated-specific primers for the *P16*, *RASSF1A*, *RPRM*, and *RUNX3* were amplified in all subjects. There was no significant association between methylation of four candidate genes and gender. In addition, stratification of subjects by ages (≤60 and >60) showed that there were no significant association between methylation of *RASSF1A*, *RPRM*, and *RUNX3* and ages. However, methylated *P16* was significantly higher in the subjects over 60 years old compared to the subjects under 60 years old (p=0.006).

**Table 2.** Methylation frequencies of the *P16, RASSF1A, RPRM,* and *RUNX3* in the subjects and their performances in detection of gastric cancer with various stages

**Gene**	**TNM stage**	**Gastric cancer (n=96)**	**Controls (n=88)**	**p-value**	**AUC**	**S**	**Sp**	**PPV**	**NPV**	**Accuracy**

		**Met (%)**	**Met (%)**							
***P16***										
	I+II	12/34 (35.3%)	14/88 (15.9%)	0.026	0.60	0.35	0.84	0.46	0.77	0.71
	III+IV	28/62 (45.2%)		p<0.001	0.65	0.45	0.84	0.67	0.69	0.68
	I–IV	40/96 (41.7%)		p<0.001	0.63	0.42	0.84	0.74	0.57	0.62
***RASSF1A***										
	I+II	8/34 (23.5%)	0/88	p<0.001 ^*^	0.62	0.24	1.0	1.0	0.77	0.79
	III+IV	24/62 (38.7%)		p<0.001	0.69	0.39	1.0	1.0	0.70	0.75
	I–IV	32/96 (33.3%)		p<0.001	0.67	0.33	1.0	1.0	0.58	0.65
***RPRM***										
	I+II	16/34 (47.1%)	6/88 (6.8%)	p<0.001	0.70	0.47	0.93	0.73	0.82	0.80
	III+IV	48/62 (77.4%)		p<0.001	0.85	0.77	0.93	0.89	0.85	0.87
	I–IV	64/96 (66.7%)		p<0.001	0.80	0.67	0.93	0.91	0.72	0.79
***RUNX3***										
	I+II	20/34 (58.8%)	4/88 (4.5%)	p<0.001	0.77	0.59	0.95	0.83	0.86	0.85
	III+IV	36/62 (58.1%)		p<0.001	0.76	0.58	0.95	0.90	0.76	0.80
	I–IV	56/96 (58.3%)		p<0.001	0.77	0.58	0.95	0.93	0.68	0.76

TNM: Tumor-Node-Metastasis; AUC: Area under the curve; S: Sensitivity; Sp: Specificity; PPV: Positive predictive value; NPV: Negative predictive value.

* Fisher’s exact tests.

**Figure 2.**
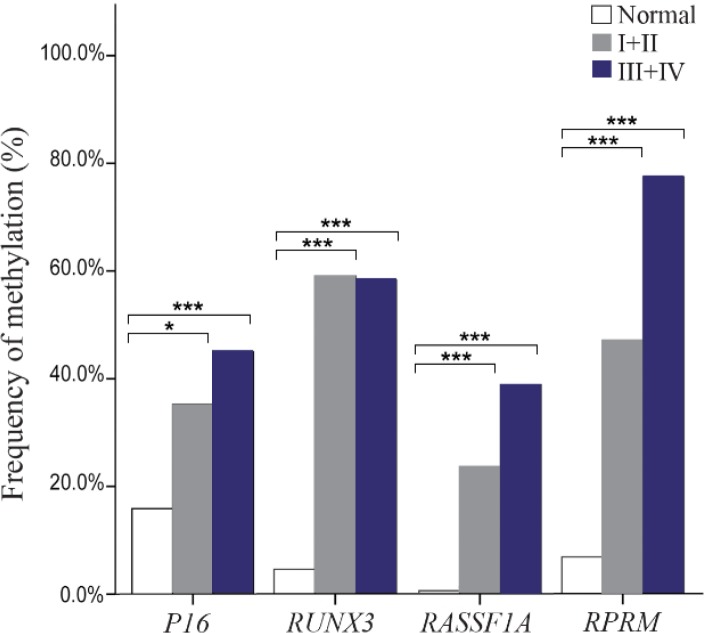
Frequency of methylated DNA in the plasma of controls and gastric cancer patients with early (I+II) and advanced-stage (III+IV). * and *** indicated p<0.05 and p<0.001, respectively.

**Figure 3.**
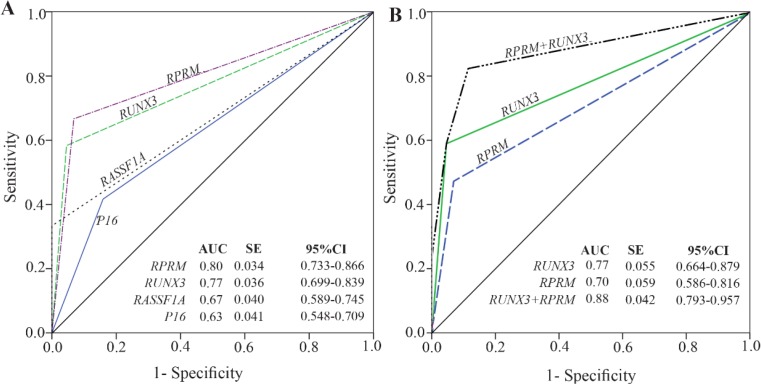
Performance of the candidate biomarkers. A) Receiver operating characteristic (ROC) for methylation status of the *P16*, *RASSF1A*, *RPRM*, and *RUNX3* in detection of gastric cancer (I–IV). B) ROC analysis of the combined *RPRM* and *RUNX3* methylation status in detection of early-stage gastric cancer (I+II). AUC: Area under curve; SE: Standard error; 95% CI: 95% Confidence interval.

Methylation rates increased in the progression of gastric carcinogenesis from the controls to the early and advanced-stages GC samples for the *P16, RASSF1A,* and *RPRM* genes (Figure 2). Concurrent methylation in two or more genes was found in 77.1% (74/96) of plasma GC samples and 0.0% of normal plasma. On the other hand, 4.2% (4/96) of GC samples and 75% (66/88) of controls were methylation free for the *P16, RASSF1A, RPRM,* and *RUNX3.* Analysis of the biomarkers performances in patients (TM stages I–IV) showed that the AUC of *RPRM*, *RUNX3*, *RASSF1A*, and *P16* were 0.80 (SE=0.034; 95% CI: 0.733–0.866), 0.77 (SE=0.036; 95% CI: 0.699–0.839), 0.67 (SE= 0.040; 95% CI: 0.589–0.745), and 0.63 (SE=0.041; 95% CI: 0.548–0.709), respectively (Figure 3).

To explore the potentials of methylation analysis of these genes in early detection of GC, the performances of *RPRM*, *RUNX3*, *RASSF1A*, and *P16* in early-stage GC patients (I+II) were analyzed in comparison to the controls. The results showed that *RUNX3* (p<0.001) with an AUC of 0.77 (SE=0.055; 95% CI: 0.664–0.879), sensitivity of 0.59, and specificity of 0.95, and *RPRM* (p<0.001) with an AUC of 0.70 (SE=0.059; 95% CI: 0.586–0.816), sensitivity of 0.47, and specificity of 0.93 could discriminate the early-stages GC patients from the normal controls with the highest performances (Table 2). Although methylation frequency of *P16* (p=0.026) and *RASSF1A* (p<0.001) was significantly different between the early-stage GC patients and the healthy people, however, they were excluded from further analysis due to their low sensitivities of 0.35 and 0.24, respectively (Table 2).

Since the sensitivity of a single gene methylation was still unsatisfying, combined detection of several genes might be a solution. A logistic regression model was performed to evaluate the diagnostic performance of the combination of the *RPRM*, and *RUNX3* biomarkers in discriminating between early-stage GC (I+ II) and normal controls (Figure 3). Our results showed that combination of *RPRM* and *RUNX3* increased the AUC to 0.88 (SE=0.042; 95% CI: 0.793–0.957), and sensitivity to 0.82; however, the specificity decreased to 0.89 (p<0.001).

### Correlations between methylation status and survival

After a median follow up period of 20 months, 16.7% (16/96) of patients died because of the disease progression. The *P16, RASSF1A*, *RPRM,* and *RUNX3* methylation were detected in 50% (8/16), 37.5% (6/16), 87.5% (14/16), and 87.5% (14/16) of these patients, respectively. Among the entire cohort, the mean survival time was 18.1 months (SE=0.422; 95% CI: 17.2–18.9).

**Figure 4.**
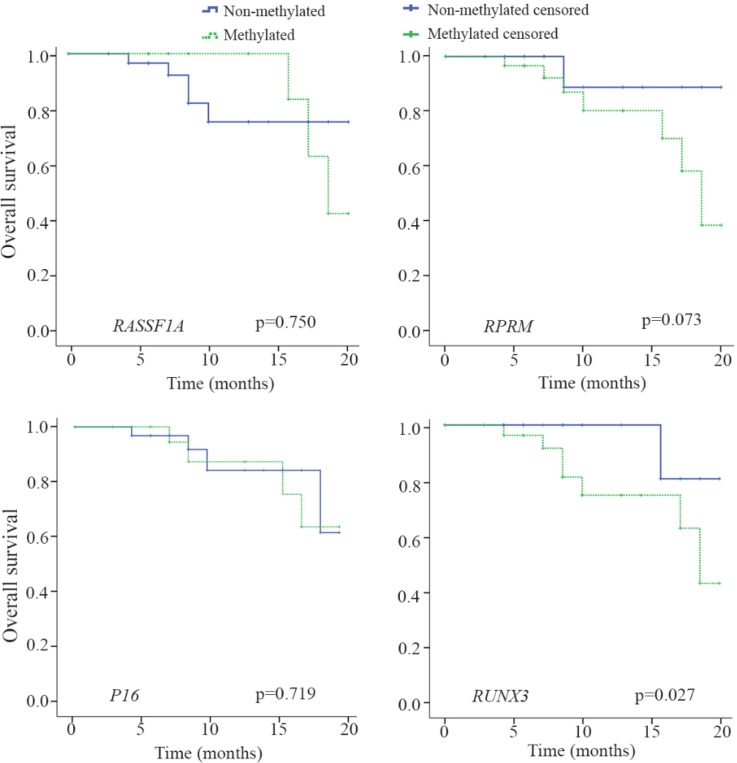
Kaplan-Meier estimate of overall survival for the gastric cancer patients with or without methylation of *P16*, *RASSF1A*, *RPRM*, and *RUNX3*. Log-rank statistics are shown as p-values.

Patients’ survival as depicted in figure 4 was significantly associated with methylation status of *RUNX3* (p=0.027). On the other hand, patients’ survival was not significantly associated with methylation status of *P16* (p=0.719), *RASSAF1A* (p=0.750), *RPRM* (p= 0.073). Mean survival time (months) of patients with a methylated *P16, RASSF1A, RPRM,* and *RUNX3* were 18.0±0.61 (95% CI: 16.8–19.2), 18.9±0.34 (95% CI: 18.2–19.6), 17.7±0.53 (95% CI: 16.6–18.7), and 17.4± 0.59 (95% CI: 16.2–18.5), respectively. In addition, mean survival time (months) of patients with an unmethylated *P16, RASSF1A, RPRM,* and *RUNX3* were 18.2±0.58 (95% CI: 17.1–19.3), 17.9±0.58 (95% CI: 16.8–19.1), 19.1±0.59 (95% CI: 17.9–20.3), and 19.4± 0.38 (95% CI: 18.7–20.1), respectively.

## Discussion

In the present study, an attempt was made to investigate the methylation status of four potential candidate genes in circulating cfDNA of plasma in GC patients and healthy controls, and their possible correlations with tumor stage, gender, age, and survival were examined. Our results showed that *P16*, *RASSF1A*, *RPRM*, and *RUNX3* promoters were methylated in 41.7, 33.3, 66.7, and 58.3% of the GC patients (I–IV), and in 15.9, 0.0, 6.8 and 4.5% of the healthy controls, respectively (p<0.001). No significant correlation was found between the methylation status of four candidate genes and gender. Analysis of methylation status with the ages (≤60 versus >60) showed that methylated *P16* was significantly higher in the subjects over 60 years old compared to the subjects under 60 years old (p=0.006). Analysis of overall survival with methylation status of the four candidate biomarkers showed that patients’ survival was significantly associated with methylation status of *RUNX3* (p=0.027).

The methylation rates in our study were similar to the results of previous studies. The results of previous studies on serum/plasma, which have evaluated the diagnostic potentials of the candidate genes methylation, are summarized in table 3. According to the previous studies by serum and plasma samples, the mean methylation rates of *P16* 23–25, *RASSF1A* 19,26,27, *RPRM* 21,22,28,29, and *RUNX3* 20,30,31 were 52.8, 61.9, 84.5, and 47.5% in the GC patients and 0.8, 1.8, 6.2, and 0.0% in the control groups, respectively. Evaluation of their findings revealed that variations in the results were lower in the controls compared to the GC groups. The methylation rates of previous studies in the GC groups for the *P16* 23,24, *RASSF1A* 26,27, *RPRM* 21,28 and *RUNX3* 30,31 ranged from 26.9–79.7, 34.0–83.2, 62.0–95.3, and 29.0–70.8%, respectively. These discrepancies could be explained by several factors. First, various methods with different sensitivity including MSP, qMSP, and bisufite sequencing have been used for analysis of methylation status of the relevant gene in each study. Second, methylation specific primers have been designed in different CpG sites for each gene. Analysis of eight CpG sites in *RUNX3* promoter by quantitative pyrosequencing has shown that methylation frequencies of only six specific sites were different between GC and normal gastric tissues 32.

**Table 3.** Methylation rates of the *P16*, RASSAF1A, *RPRM*, and *RUNX3* in diagnosis of gastric cancer using serum or plasma samples

**Gene**	**Source**	**Gastric cancer**	**Control**	**Country**	**Method**	**Reference**
***P16***						
	Serum	51.9%	0.0%	Hong Kong	MSP	25
	Serum	26.9%	0.0%	Iran	MSP	24
	Serum	79.7%	2.5%	China	MSP	23
	Plasma	41.7%	15.9%	Iran	MSP	This Study
***RASSF1A***						
	Serum	34.0%	0.0%	China	MSP	27
	Serum	68.5%	0.0%	Greece	MSP	19
	Plasma	83.2%	5.5%	Thailand	MSP	26
	Plasma	33.3%	0.0%	Iran	MSP	This Study
***RPRM***						
	Plasma	95.3%	9.7%	Chile	MSP	28
	Plasma	62.0%%	0.0%	China	MSP	21
	Serum	94.3%	7.1%	China	MS-MCA	22
	Plasma	86.3%	7.9%	China	BS	29
	Plasma	66.7%	6.8%	Iran	MSP	This Study
***RUNX3***						
	Serum	29.0%	0.0%	Japan	qMSP	31
	Serum	70.8%	0.0%	China	qMSP	30
	Plasma	42.7%	0.0%	China	MSP	20
	Plasma	58.3%	4.5%	Iran	MSP	This Study

MS-MCA: Methylation sensitive melt curve analysis; MSP: Methylation specific PCR; q-MSP: quantitative MSP; BS: Bisufite sequencing.

Our results showed that the methylation rates increased in the progression of gastric carcinogenesis from the control to the early and advanced-stage GC for the *P16*, *RASSF1A*, and *RPRM* genes. Increasing the methylation rate could be explained by the fact that more cfDNA gets into systemic circulation with enhancement of the disease. In addition, concurrent methylations in two or more genes were found in 77.1% of plasma GC samples and 0.0% of normal plasma. On the other hand, 4.2% of GC patients and 75.0% of controls were methylation free for all four genes.

Also, the performances of candidate biomarkers in early-stage GC patients (I+II) were analyzed. The results showed that *RUNX3* with an AUC of 0.77, sensitivity of 0.59, and specificity of 0.95, and *RPRM* with an AUC of 0.70, sensitivity of 0.47, and specificity of 0.93 could discriminate the early-stage GC from normal controls with the highest performances (p<0.001). Although *P16* (p=0.026) and *RASSF1A* (p<0.001) could also discriminate the early-stage patients from the controls, their sensitivities were very low as 0.35 and 0.24, respectively. For that reason, they were excluded from further analysis.

The sensitivity and specificity of *RUNX3* hypermethylation by qMSP, in serum samples of GC patients and normal controls in a study by Lu *et al* were 70.8 and 99.8%, respectively 30. In another study by Lin *et al*, the sensitivity and specificity of *RUNX3* hypermethylation were 42.7 and 79.2%, respectively 20. Our results were similar to the study by Lin *et al* which used MSP as the method for methylation detection 20. The higher performance of *RUNX3* methylation in the study by Lu *et al* might be explained by the fact that qMSP is more sensitive than MSP 30.

The use of only a single gene to discriminate cancer patients from the healthy people has several drawbacks. First, the maximum sensitivity of a test by only a single gene could be as high as the rate of methylation for that gene. Second, non-cancerous tissues could be occasionally methylated at the same gene locus as cancerous tissue 33. Furthermore, methylation of a single gene locus can occur in different cancers. For example, in addition to GC, aberrant methylation of *P16* and *RASSF1A* in serum of breast cancer patients 34, hypermethylation of *RASSF1A* in hepatocellular carcinoma tissues 35, and hypermethylation of *RUNX3* in serum of colorectal cancer patients have also been reported 36. For that reason, a panel of hypermethylated genes, instead of a single gene, could be more effective to guarantee that the biomarker is specific to a cancer.

Also, the combination of *RPRM* and *RUNX3* in distinguishing the early-stage GC (I+II) from the controls was analyzed. The results showed a higher AUC of 0.88, with a higher sensitivity of 0.82 and reduced specificity of 0.89 (p<0.001). Although the specificity of combined detection of *RPRM* and *RUNX3* methylation was lower than that of single gene assays, the sensitivity was increased.

## Conclusion

In conclusion, combined detection of plasma *RPRM*, and *RUNX3* methylation could be suggested as a potential strategy for early diagnosis of GC; however, further studies for validation of the panel are required.
